# Confidence Interval Construction for Causally Generalized Estimates With Target Sample Summary Information

**DOI:** 10.1002/sim.70358

**Published:** 2026-01-22

**Authors:** Yi Chen, Guanhua Chen, Menggang Yu

**Affiliations:** ^1^ Department of Biostatistics and Medical Informatics University of Wisconsin Madison Wisconsin USA; ^2^ Department of Biostatistics University of Michigan Ann Arbor Michigan USA

**Keywords:** causal generalization, confidence interval, entropy balancing weights, resampling‐based perturbation, summary‐level data

## Abstract

Generalizing causal findings, such as the average treatment effect (ATE), from a source to a target population is a critical topic in biomedical research. Differences in the distributions of treatment effect modifiers between these populations, known as covariate shift, can lead to varying ATEs. Chen et al. [1] introduced a weighting method to estimate the target ATE using only summary‐level information from a target sample while accounting for the possible covariate shifts. However, the asymptotic variance of the estimate was shown to depend on individual‐level data from the target sample, hindering statistical inference. In this article, we propose a resampling‐based perturbation method for confidence interval construction for the estimated target ATE, utilizing additional summary‐level information. We demonstrate the effectiveness of our approach through simulation and real data settings when only summary‐level information is available.

## 1 Introduction

Causal inference plays a pivotal role in population health research, providing essential tools for understanding and shaping effective health interventions. One of its popular research questions is how to generalize causal findings from a study population to a target population [1, 2, 3]. For example, we may want to generalize findings about the effectiveness of a treatment from a properly conducted randomized clinical trial (RCT) to its target population. We usually refer to this type of problem as generalizability [4, 5], transportability [6, 7], or data fusion [8, 9, 10]. There are some differences between these terminologies, and more detailed explanations are found in article by Colnet et al. [3].

For much of this article, for demonstration purposes, we focus on generalizing the average treatment effect (ATE), although similar considerations can be given to other causal estimands such as the Average Treatment effect on the Treated (ATT) or Average Treatment effect on the Overlap population (ATO) [3]. Our method mainly deals with causal generalization from a source to a target population when individual treatment effects are heterogenenous. Specifically, the individual treatment effects may depend on certain covariates, known as effect modifiers. In addition, the distributions of the effect modifiers can differ between the two populations [11].

Much of the existing literature take a data fusion or integrated data analysis approach to this problem [3, 8, 9, 10, 12]. Such approaches typically require individual data from both populations. However, there can be settings when comprehensive data at the individual level may not be consistently accessible within a target sample, owing to various practical considerations such as restricted data sharing, storage constraints, and privacy apprehensions [2]. On the contrary, obtaining summary‐level information from the target sample is comparatively more feasible. This type of information can be readily gathered from diverse sources such as healthcare databases, census data, and published literature.

To deal with the challenges posed by lack of individual data from the target population, Dong et al. [13] adapted the entropy balancing weights approach [14, 15] for generalizing ATE estimation from an RCT to a given target population. Josey et al. [16] then extended the approach to the setting when the source sample is from observational studies. In particular, they proposed a two‐step procedure to adjust for covariate shift and confounding separately. By showing that the weights produced by the two‐step procedure can be consolidated into a one‐step procedure, Chen et al. [1] developed a more intuitive strategy that may further mitigate bias under mild conditions, which rely solely on summary‐level information from the target sample and individual‐level covariates from the source sample. Recently, Chattopadhyay et al. [17] proposed a very similar strategy.

The purpose of this article is to provide a practical solution to a key limitation with these methods: how to construct confidence intervals (CIs) for the resulting causally generalizable estimates. Chen et al. [1] showed that the asymptotic variance of their estimator depends on individual‐level data in the target sample. Similarly, the asymptotic variance of the estimator from Chattopadhyay et al. [17] also depends on the individual‐level data in the target sample. This article addresses this limitation by proposing a method to construct CIs for the proposed estimator from Chen et al. [1] using resampling‐based perturbation, without requiring individual‐level data from the target sample.

This article is organized as follows: in Section 2, we present general notations and assumptions for our method. In Section 3, we present two methods to do the resampling‐based perturbation for CI construction. In Sections 4 and 5, we evaluate the proposed methods using simulation studies and a real data application using cross‐validation. In Section 6, we conclude the article with a discussion.

## 2 Notation and Framework

Suppose we have individual‐level data in a representative sample of our source population 𝒮, denoted as $\left\{(X_{i};A_{i};Y_{i}):i\in 𝒮\right\}$ with $n_{s}$ subjects. We denote $X_{i}\in 𝒳\subset {\mathbb{R}}^{p}$ as the pre‐treatment covariates which include confounders and treatment effect modifiers. The treatment indicator is denoted as $A_{i}\in \{0,1\}$, and $Y_{i}$ is the outcome we are interested in. For a representative sample of our target population 𝒯, the sample size is $n_{t}$ but we do not observe the individual‐level data. Instead, we only have the information for the first moments based on a set of linearly independent covariate functions $h_{k}:𝒳\to \mathbb{R};k=1,\ldots ,K_{h}$ from the target sample as follows.

$${\bar{h}}_{k,𝒯}\equiv \frac{1}{n_{t}}\underset{i\in 𝒯}{\sum }h_{k}(X_{i}),k=1,\ldots ,K_{h}\;.$$

Each $h_{k}$ is usually defined on one or two covariates, instead of on the full covariate vector. For continuous covariates, if $h_{k}$ is defined as an identity function, then ${\bar{h}}_{k,𝒯}$ represents the mean of this component. If $h_{k}$ is defined as a polynomial function of degree 2, ${\bar{h}}_{k,𝒯}$ corresponds to the second moment, or variance, of this component. For discrete covariates, $h_{k}$ could be defined as an indicator function to count the number of subjects in a particular category.

Here, we formulate the causal problem using the potential outcome framework [18, 19]. For each subject, we define a “full” random vector $\left(X_{i},S_{i},A_{i},Y_{i}(0),Y_{i}(1)\right)$, where $S_{i}$ is a population indicator in source or target such that $S_{i}=1$ for i∈𝒮 and $S_{i}=0$ for i∈𝒯. The total sample size is $n=n_{s}+n_{t}$ and each subject assumed to be i.i.d. from a joint distribution of (X,S,A,Y(0),Y(1)). Moreover, ${𝒮}_{0}$ is used to denote the subjects in the source control group, and mathematically, ${𝒮}_{0}=\{i:S_{i}=1;A_{i}=0\}$; ${𝒮}_{1}$ is defined for the source treated group similarly. According to Rosenbaum and Rubin [19], we use the propensity score π(x)=ℙ(A=1|X=x,S=1) to determine the treatment assignment mechanism. The main estimand in this article, ATE of the target population, is

(1) $$\begin{array}{l} \tau^{*}=𝔼\{Y(1)-Y(0)|S=0\}, \end{array}$$

The following 3 standard assumptions are used which enable identification of causal effects within the source population.

Assumption 1 (Stable Unit Treatment Value Assumption or SUTVA) There is no interference between different subjects and no hidden variation of treatments.

Assumption 2 (No unmeasured confounders of treatment assignment) In the source population, (Y(0),Y(1)) are conditionally independent of A given X: (Y(0),Y(1))╨A|X,S=1.

Assumption 3 (Positivity of treatment assignment) The propensity score of the source population is bounded away from 0 and 1: for some c>0, c≤π(X)≤1−c almost surely.

To extend the generalizability of the causal estimates to the target population, a key quantity is the participation probability between source and target defined as ρ(x)=ℙ(S=1|X=x). We further adopt two additional assumptions from Rudolph and van der Laan [6] and Dahabreh et al. [20].

Assumption 4 (Mean exchangeability across populations) The conditional mean of the potential outcomes given the covariates are equal between the two populations: 𝔼{Y(a)|X,S=1}=𝔼{Y(a)|X,S=0} almost surely for a∈{0,1}.

Assumption 5 (Positivity of participation probability) The participation probability is bounded away from 0: ρ(X)>c almost surely for some c>0.

We further denote the conditional mean and variance of the potential outcomes in the source population as $\mu_{a}(x)=𝔼\{Y(a)|X=x,S=1\}$ and $\sigma_{a}(x)=\mathrm{Var}\{Y(a)|X=x,S=1\}$. Under Assumption 4, we have $\mu_{a}(x)=𝔼\{Y(a)|X=x,S=0\}=𝔼\{Y(a)|X=x\}$. The conditional average treatment effect (CATE) function is denoted as $\tau (x)\equiv \mu_{1}(x)-\mu_{0}(x)$.

## 3 Method

### 3.1 Gap in the Existing Work

Given Assumptions 1, 2, 3, 4, 5, we can estimate $\tau^{*}$ in terms of the observable from the source sample data by a difference of weighted outcomes as follows:

(2) $$\begin{array}{l} {\hat{\tau }}_{w}=\frac{1}{n_{s}}\underset{i\in {𝒮}_{1}}{\sum }w_{i}Y_{i}-\frac{1}{n_{s}}\underset{i\in {𝒮}_{0}}{\sum }w_{i}Y_{i}\;. \end{array}$$

The weights $\left\{w_{i}:i\in 𝒮\right\}$ take the following form Chen et al. [1] and in appendix, we also provide the identification proof:

$$w_{i}=\left\{\frac{A_{i}}{\pi (X_{i})}+\frac{1-A_{i}}{1-\pi (X_{i})}\right\}\frac{E(S_{i})(1-\rho (X_{i}))}{\left(1-E(S_{i}))\rho (X_{i}\right)}.$$

Directly estimation of $w_{i}$ is usually computationally unstable. Without the individual data it is also infeasible. Therefore, Chen et al. [1] proposed a method for estimation of the weights as follows, based on entropy balancing weighting framework [14].

(3) $$\begin{array}{ll} \underset{w⪰0}{\min} & \underset{i\in 𝒮}{\sum }w_{i}\log w_{i}\\ \text{subject to}\;\; & \frac{1}{n_{s}}\underset{i\in {𝒮}_{1}}{\sum }w_{i}h_{k}(X_{i})={\bar{h}}_{k,𝒯},\;k=1,\ldots ,K_{h};\\ & \frac{1}{n_{s}}\underset{i\in {𝒮}_{0}}{\sum }w_{i}h_{k}(X_{i})={\bar{h}}_{k,𝒯},\;k=1,\ldots ,K_{h};\\ & \frac{1}{n_{s}}\underset{i\in {𝒮}_{1}}{\sum }w_{i}g_{k}(X_{i})=\frac{1}{n_{s}}\underset{i\in {𝒮}_{0}}{\sum }w_{i}g_{k}(X_{i}),\;k=1,\ldots ,K_{g};\\ & \frac{1}{n_{s}}\underset{i\in {𝒮}_{1}}{\sum }w_{i}=\frac{1}{n_{s}}\underset{i\in {𝒮}_{0}}{\sum }w_{i}=1. \end{array}$$

In particular, functions $\left\{h_{k}:X\to \mathbb{R};k=1,\ldots ,K_{h}\right\}$ are used to address covariate shift between source and target samples, while functions $\left\{g_{k}:X\to \mathbb{R};k=1,\ldots ,K_{g}\right\}$ are employed to further correct for imbalances between the treatment and control groups within the source sample. From the theorem below, we can see that ideally the $h_{k}$ functions should be chosen so that the linear span formed by them can at least cover treatment modifiers, even all outcome related variables if possible. The $g_{k}$ functions should be chosen to complement $h_{k}$ to determine the treatment assignment mechanism.

The weight normalization constraint at the last line of Equation (3) can be absorbed to the first two constraints by introducing $h_{0}(x)\equiv 1$. Denote $H=(h_{0},h_{1},\ldots ,h_{K_{h}})$ and $G=(g_{1},\ldots ,g_{K_{g}})$. The following theorem is adopted directly from Chen et al. [1] which originally listed 3 conditions under any of which could lead to consistency of the resulting weighting estimator for $\tau^{*}$. Here we only list two of them as the other one was not as intuitive.

Theorem 1 *Suppose* ŵ *is the solution of Equation *(*3*). *If either Condition* (*a*) *or* (*b*) *below holds*, ${\hat{\tau }}_{ŵ}$ *is a consistent estimator of* $\tau^{*}$:

*Condition* (*a*). $\mu_{a}(x)\in$ *Span* {H(x)}, a=0,1.

*Condition* (*b*). log{π(x)/(1−π(x))}∈ *Span* {H(x),G(x)} *and* τ(x)∈ *Span* ({H(x)}).

Chen et al. [1] further derived the asymptotic variance for ${\hat{\tau }}_{w}$. However, estimation of the asymptotic variance directly from their formula requires individual covariate values in the target sample. We intend to overcome this limitation by introducing a resampling‐based perturbation method for CI construction that do not require such information from the target sample.

### 3.2 Resampling‐Based Perturbation for Confidence Interval Construction

Parzen et al. [21] introduced a straightforward resampling method for inference based on pivotal estimating functions within a semiparametric model framework. The authors demonstrated that for a broad class of estimating functions meeting two mild convergence conditions, a valid asymptotic CI could be constructed using the resampling method on the pivotal estimating functions. Hu and Kalbfleisch [22] further broadened the idea by using bootstrapped general estimating functions for statistical inference. In particular, when the estimating functions are sums of independent terms, we can resample or bootstrap these terms to obtain an empirical distribution of the estimating functions. Solving the corresponding bootstrapped estimation equations then leads to valid statistical inference for the resulting estimators. Here, we extend this idea to our setting.

Since Equation (3) has constraints, we work with its dual problem which is unconstrained for our purpose. In particular, we have the following characterization of the weights from Equation (3).

$$\begin{array}{l} {ŵ}_{i}=\{\begin{array}{cc} \exp\{{\hat{\lambda }}_{1}^{⊤}H(X_{i})+{\hat{\gamma }}^{⊤}G(X_{i})\}, & i\in {𝒮}_{1}\\ \exp\{{\hat{\lambda }}_{0}^{⊤}H(X_{i})-{\hat{\gamma }}^{⊤}G(X_{i})\}, & i\in {𝒮}_{0} \end{array} \end{array}$$

where $({\hat{\lambda }}_{1},{\hat{\lambda }}_{0},\hat{\gamma })\in {\mathbb{R}}^{K_{h}+1}\times {\mathbb{R}}^{K_{h}+1}\times {\mathbb{R}}^{K_{g}}$ is the solution to the dual problem:

(4) $$\begin{array}{ll} & \underset{\lambda_{1},\lambda_{0},\gamma }{\min}\;\frac{1}{n_{s}}\underset{i\in {𝒮}_{1}}{\sum }\exp\{\lambda_{1}^{⊤}H(X_{i})+\gamma^{⊤}G(X_{i})\}\\ & \;+\frac{1}{n_{s}}\underset{i\in {𝒮}_{0}}{\sum }\exp\{\lambda_{0}^{⊤}H(X_{i})-\gamma^{⊤}G(X_{i})\}\\ & \;-(\lambda_{1}^{⊤}+\lambda_{0}^{⊤}){\bar{H}}_{𝒯}. \end{array}$$

Here ${\bar{H}}_{𝒯}=({\bar{h}}_{0,𝒯},\ldots ,{\bar{h}}_{K_{h},𝒯})$ with ${\bar{h}}_{0,𝒯}=1$.

Equation (4) leads to the following first order condition to solve for $\left({\hat{\lambda }}_{1},{\hat{\lambda }}_{0},\hat{\gamma }\right)$:

(5) $$\begin{array}{l} \begin{array}{cc} & {n_{s}}^{-1}\underset{i\in S_{1}}{\sum }H(X_{i})\exp\{\lambda_{1}^{⊤}H(X_{i})+\gamma^{⊤}G(X_{i})\}-{\bar{H}}_{T}=0\\ & {n_{s}}^{-1}\underset{i\in S_{0}}{\sum }H(X_{i})\exp\{\lambda_{0}^{⊤}H(X_{i})-\gamma^{⊤}G(X_{i})\}-{\bar{H}}_{T}=0\\ & \underset{i\in S_{1}}{\sum }G(X_{i})\exp\{\lambda_{1}^{⊤}H(X_{i})+\gamma^{⊤}G(X_{i})\}\;\\ & \;-\underset{i\in S_{0}}{\sum }G(X_{i})\exp\{\lambda_{0}^{⊤}H(X_{i})-\gamma^{⊤}G(X_{i})\}=0\; \end{array} \end{array}$$

Therefore, if we can use bootstrap to capture the variance of the estimating equations in Equation (5), we can back‐propagate the estimation error to the estimated weights, enabling us to construct a CI for the estimator ${\hat{\tau }}_{w}$ in Equation (2). The classic bootstrap [23] can be applied to the elements in the source population $\left\{H(X_{i}),G(X_{i})\right\}$ to generate bootstrapped versions $\left\{H{\left(X_{i}\right)}^{\left(b\right)},G{\left(X_{i}\right)}^{\left(b\right)}\right\}$ for b=1,…,B. However, for the summary level information ${\bar{H}}_{𝒯}$, we resort to parametric bootstrap [24]. Because ${\bar{H}}_{𝒯}$ are sample averages, we assume that ${\bar{H}}_{𝒯}∼N(\mu_{\bar{H}},\sum_{\bar{H}})$ asymptotically. Therefore, when $\sum_{\bar{H}}$ is available from the target sample, we can draw ${\bar{H}}_{𝒯}^{\left(b\right)}$ from the multivariate normal distribution with mean ${\bar{H}}_{𝒯}$ and variance‐covariance matrix $\sum_{\bar{H}}$. It is more common that only the diagonal elements of $\sum_{\bar{H}}$ is available, especially if the summary information is from published literature. Then we propose to estimate the correlation matrix corresponding to $\sum_{\bar{H}}$ using the individual data from the source population.

We formalize the above proposed resampling‐based perturbation method to construct the confidence interval (RPM‐CI) in Algorithm 1. For a particular data set, in step 1, we estimate the correlation of target moments ${\hat{R}}_{{\bar{H}}_{𝒯}}=\text{corr}(H(X_{i})),i\in 𝒮$ using the source data and estimate the corresponding target ATE ${\hat{\tau }}_{w}$. In step 2, for each $b^{th}$ over B iteration, we first sample the source population with replacement as $\left\{(X_{i}^{\left(b\right)};A_{i}^{\left(b\right)};Y_{i}^{\left(b\right)}):i\in 𝒮\right\}$. Then we use multivariate normal distribution to perturb the target sample mean ${\bar{H}}_{𝒯}$ and generate target data perturbed means ${\bar{H}}_{𝒯}^{\left(b\right)}∼𝒩({\bar{H}}_{𝒯},\mathrm{var}{\left({\bar{H}}_{𝒯}\right)}^{1/2}{\hat{R}}_{{\bar{H}}_{𝒯}}\mathrm{var}{\left({\bar{H}}_{𝒯}\right)}^{1/2})$ assuming $\mathrm{var}({\bar{H}}_{𝒯})$ is available from the target sample. Next, we use the simulated $\left\{(X_{i}^{\left(b\right)};A_{i}^{\left(b\right)};Y_{i}^{\left(b\right)}):i\in 𝒮\right\}$ and ${\bar{H}}_{𝒯}^{\left(b\right)}$ to estimate weights $\left\{{ŵ}_{i}^{\left(b\right)}:i\in S\right\}$ and its corresponding ${\hat{\tau }}_{w}^{\left(b\right)}$ for b=1,…,B using Equation (3) and (2). Finally, we construct the CI for our estimator ${\hat{\tau }}_{w}$ based on 2.5 and 97.5 percentiles of ${\hat{\tau }}_{w}^{\left(b\right)},b=1,\ldots ,B$.

ALGORITHM 1 **Resampling‐based perturbation method for CI construction (RPM‐CI).**

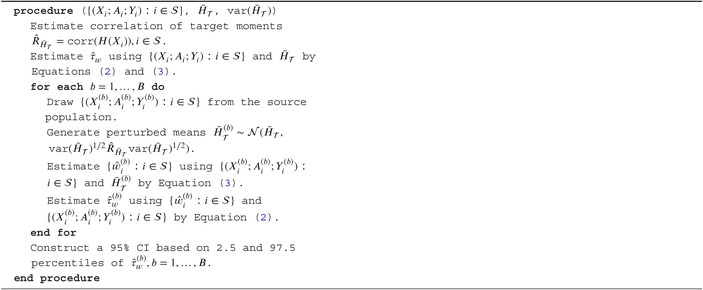

### 3.3 Resampling‐Based Perturbation Method With Approximate Balancing

In practice, the exact balancing approach may not always produce a feasible solution due to finite sample. Therefore, Wang and Zubizarreta [25] advocated a more flexible approach for covariate balancing weight construction for causal inference. The approach can be extended in a straight‐forward fashion to our causal generalization setting as follows.

(6) $$\begin{array}{ll} \underset{w_{i}⪰0}{\min}\;\; & \underset{i\in S}{\sum }w_{i}\log w_{i}\\ \text{subject to}\;\; & |\frac{1}{n_{s}}\underset{i\in {𝒮}_{1}}{\sum }w_{i}h_{k}(X_{i})-{\bar{h}}_{k,𝒯}|\leq \delta_{1},k=1,\ldots ,K_{h};\\ & |\frac{1}{n_{s}}\underset{i\in {𝒮}_{0}}{\sum }w_{i}h_{k}(X_{i})-{\bar{h}}_{k,𝒯}|\leq \delta_{1}^{\prime },k=1,\ldots ,K_{h};\\ & |\frac{1}{n_{s}}\underset{i\in {𝒮}_{1}}{\sum }w_{i}g_{k}(X_{i})-\frac{1}{n_{s}}\underset{i\in {𝒮}_{0}}{\sum }w_{i}g_{k}(X_{i})|\leq \delta_{2},k=1,\ldots ,K_{g};\\ & \frac{1}{n_{s}}\underset{i\in {𝒮}_{1}}{\sum }w_{i}=\frac{1}{n_{s}}\underset{i\in {𝒮}_{0}}{\sum }w_{i}=1. \end{array}$$

Therefore, the exact balancing constraints is relaxed in Equation (6) by introducing $\delta_{1},\delta_{1}^{\prime }\in {\mathbb{R}}^{K_{h}}$ and $\delta_{2}\in {\mathbb{R}}^{K_{g}}$. The weight normalization constraint at the last line of Equation (6) again can be absorbed to the first two constraints by introducing an extra element ${\bar{h}}_{0,𝒯}=1$ and setting the corresponding relaxation $\delta_{1,0},\delta_{1,0}^{\prime }\equiv 0$. This flexibility trades bias for variance and offers two key advantages: it enables us to incorporate a broader set of covariate functions, and it helps overcome computational challenges when exact balancing is infeasible during the construction of CIs with the resampling‐based perturbation.

Naturally, a practical consideration when using approximate balancing is how to determine the appropriate degree of approximate balance. Chattopadhyay et al. [17] advocated using a constant factor (i.e., 0.1 times) of each covariate's standard deviation. Instead of the standard deviation, we advocate the following strategy based on the dual problem of Equation (6).

In particular, the dual of Equation (6) takes the following form:

$${\tilde{w}}_{i}=\{\begin{array}{ll} \exp\{{\tilde{\lambda }}_{1}^{⊤}H(X_{i})+{\tilde{\gamma }}^{⊤}G(X_{i})\}, & i\in {𝒮}_{1}\\ \exp\{{\tilde{\lambda }}_{0}^{⊤}H(X_{i})-{\tilde{\gamma }}^{⊤}G(X_{i})\}, & i\in {𝒮}_{0} \end{array}$$

where ${\tilde{\lambda }}_{0},{\tilde{\lambda }}_{1},\tilde{\gamma }$ minimize

(7) $$\begin{array}{ll} & \underset{\lambda_{1},\lambda_{0},\gamma }{\min}\;\frac{1}{n_{s}}\underset{i\in {𝒮}_{1}}{\sum }\exp\{\lambda_{1}^{⊤}H(X_{i})+\gamma^{⊤}G(X_{i})\}\\ & \;+\frac{1}{n_{s}}\underset{i\in {𝒮}_{0}}{\sum }\exp\{\lambda_{0}^{⊤}H(X_{i})-\gamma^{⊤}G(X_{i})\}\\ & \;-\lambda_{1}^{⊤}{\bar{H}}_{𝒯}-\lambda_{0}^{⊤}{\bar{H}}_{𝒯}+|\lambda_{1}{|}^{⊤}\delta_{1}+|\lambda_{0}{|}^{⊤}\delta_{1}^{\prime }+|\gamma {|}^{⊤}\delta_{2}. \end{array}$$

Compared with the dual form (4) for the exact balancing, Equation (7) contains three additional $L_{1}$ regularization terms for the dual parameters: $|\lambda_{1}{|}^{⊤}\delta_{1}+|\lambda_{0}{|}^{⊤}\delta_{1}^{\prime }+|\gamma {|}^{⊤}\delta_{2}$. Thus, we propose to use the Adaptive LASSO [26] to determine the degree of approximate balancing. In particular, assume that we have estimates $\left({\hat{\lambda }}_{1},{\hat{\lambda }}_{0},\hat{\gamma }\right)$ from Equation (4) based on the exact balancing problem. Then for resampling‐based perturbations that have no exact balancing solution or are infeasible for (3), we use fractions of $\left({\hat{\lambda }}_{1},{\hat{\lambda }}_{0},\hat{\gamma }\right)$ for $\left(\delta_{1},\delta_{1}^{\prime },\delta_{2}\right)$. The details are listed in Algorithm 2.

When there is no exact balancing solution for the original exact balancing problem ${\hat{\tau }}_{w}$, we advocate using Chattopadhyay et al. [17] idea of allowing imbalances to be up to a constant factor (i.e., 0.1 times) each covariate's standard deviation until we get a solution and then follow Algorithm 2 for CI construction.

ALGORITHM 2 **Resampling‐based perturbation with approximate balancing (RPM‐AB).**

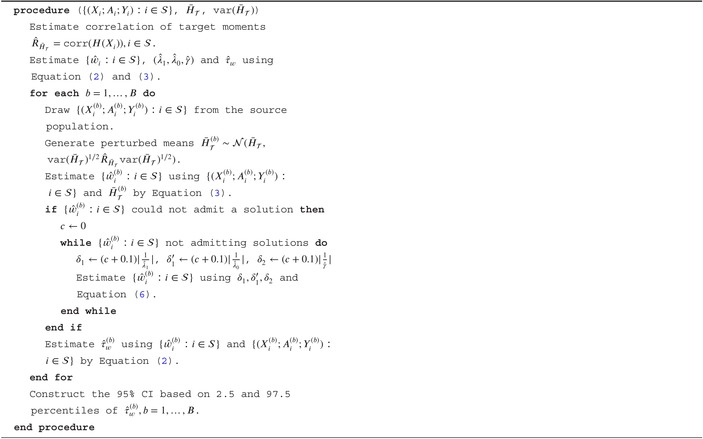

## 4 Evaluation With Simulated Settings

In this section, we conduct simulation studies to evaluate the performance of the proposed methods in finite sample settings. For each simulation set‐up, we could estimate $\tau^{*}$ using Equation (1). Then, for each m over a total of M simulations, we estimate ${\hat{\tau }}_{w}^{\left(m\right)}$ and its corresponding 95% CI using methods we proposed in Algorithm 1, or Algorithm 2 if exact balancing is infeasible. The performance is measured in terms of bias of $E({\hat{\tau }}_{w}^{\left(m\right)})$ and the empirical coverage of $\tau^{*}$ within the 95% CI constructed by the proposed methods.

### 4.1 Exact Balancing and Algorithm 1 Evaluation

We first examine exact balancing CI construction method in Algorithm 1 with simulation settings that always had feasible solutions for Equation (3) and the corresponding resampling‐based perturbations. We set the total sample size $n=n_{s}+n_{t}=800$ with the bootstrap iteration B=1000 and M=500 simulated data sets. In our simulations, due to random sampling, the source sample size $n_{s}$ varies between 350 and 450 observations. We generate 5 covariates $X=(X_{1},\ldots ,X_{5})$ from a uniform distribution U(−2,2). We consider the case when the covariates are independent of each other and the case when correlation among them are 0.1 and 0.3.

In the target sample, we only have summary‐level information of $X_{1},X_{2},$ and $X_{3}$. We set $H(x)=(1,x_{1},x_{2},x_{3})$ and $G(x)=(x_{4},x_{5})$. We consider balancing on the first moments of all covariates.

In light of Theorem 1, we consider scenarios when one of the conditions for consistency hold and also when none of them holds.

Therefore, for the propensity score model, we first assume a scenario when the treatment assignment is related to H linearly with $\text{logit}\{\pi (x)\}=0.7x_{2}+0.5x_{3}$. In this case, all the confounders are included in H, and it is enough that we only balance on H to account for confounding. We also assume a scenario when the propensity score is related to H and G nonlinearly with $\text{logit}\{\pi (x)\}=0.35x_{2}-0.4\max(x_{3},x_{4})-0.7x_{5}$.

For the outcome model, we assume it has the form of $Y_{i}=m(X_{i})+(A_{i}-0.5)\tau (X_{i})+\epsilon_{i}$ with $\epsilon_{i}\overset{\text{i.i.d}}{∼}N(0,1)$. We assume the CATE function comes from the following settings:

(T1) $\tau (x)=x_{1}-0.6x_{2}-0.4x_{3}$.
(T2) $\tau (x)=x_{1}-0.6x_{2}-0.4x_{3}+0.8x_{4}-0.3x_{5}$.
(T3) $\tau (x)=x_{1}-0.5\exp(x_{2}-0.8x_{3})$

We assume the main effect m(x) comes from the following settings:

(M1) $m(x)=0.5x_{1}+0.3x_{2}+0.3x_{3}$.
(M2) $m(x)=0.5x_{1}+0.3x_{2}+0.3x_{3}-0.4x_{4}-0.7x_{5}$.
(M3) $m(x)=0.5x_{1}+0.8x_{2}^{2}+0.2\exp(0.5x_{3}-x_{4}-1)-0.7x_{5}$.

When the propensity score lies within the linear span of H and the CATE function follows (T1), τ(x) is linearly related to H and satisfies the consistency Condition (b) in Theorem 1, regardless of the main effect settings. However, this condition does not hold under (T2) or (T3) because (T2) depends on both H and G, while (T3) is nonlinearly related to H. When the propensity score is not within the linear span of H, but the outcome satisfies (T1) and (M1), $\mu_{a}(x)$ remains linear in H, thereby meeting Condition (a). Outside these scenarios, neither Condition (a) nor Condition (b) holds, which may introduce bias in the estimator ${\hat{\tau }}_{w}$.

For covariate shift, similar to the propensity score model, we also consider a linear setting when the participation probability is $\text{logit}\{\rho (x)\}=0.4x_{1}+0.3x_{2}-0.2x_{4}$. That is, there is shift in the distribution of $\left(X_{1},X_{2},X_{4}\right)$. We also consider a nonlinear setup when the participation probability is $\text{logit}\{\rho (x)\}=0.3x_{1}+0.5x_{2}\cdot x_{4}-0.2x_{4}$.

The performance of our method is summarized in Table 1 for independent covariates and Table 2 for correlated covariates. For bias evaluation, we see that the bias of average ${\hat{\tau }}_{w}$ for our method is ignorable under linear and nonlinear settings when the consistency conditions are met. In terms of the CI construction, when the consistency conditions are met, we find that under both linear and nonlinear settings, the constructed CI by Algorithm 1 can cover around 95% of the time. Even when the consistency conditions are not fully met, our method maintains approximately 95% coverage as long as the estimator is not severely biased. However, when the estimator exhibits significant bias, the constructed confidence interval (CI) results in lower coverage of $\tau^{*}$.

**TABLE 1:** Empirical evaluation for target ATE estimation and CI coverage using the RPM‐CI method (independent covariates).

Settings		Consistency Condition	$\tau^{*}$	Empirical coverage of $\tau^{*}$	Average ${\hat{\tau }}_{w}$ (95% CI)
Linear	T1+M2	(b)	−0.140	95.2%	−0.131 (−0.422, 0.159)
	T1+M3	(b)	−0.138	95.6%	−0.152 (−0.534, 0.220)
	T2+M1	No	−0.042	76%	−0.242 (−0.561, −0.076)
Nonlinear	T1+M1	(a)	−0.179	95.5%	−0.180 (−0.570, 0.206)
	T3+M1	No	−1.525	94.2%	−1.496 (−1.843, −1.164)
	T1+M3	No	−0.179	93.2%	−0.188 (−0.538, 0.157)

**TABLE 2:** Empirical evaluation for target ATE estimation and CI coverage using the RPM‐CI method (correlated covariates).

Settings		Consistency Condition	$\tau^{*}$	Covariate correlation	Empirical coverage of $\tau^{*}$	Average ${\hat{\tau }}_{w}$ (95% CI)
Linear	T1+M2	(b)	−0.126	0.1	94.8%	−0.122 (−0.410, 0.164)
			−0.097	0.3	94.5%	−0.096 (−0.382, 0.190)
	T2+M1	No	−0.054	0.1	76.8%	−0.249 (−0.566, 0.066)
			−0.079	0.3	79.8%	−0.251 (−0.560, 0.055)
Nonlinear	T1+M1	(a)	−0.166	0.1	94%	−0.175 (−0.437, −0.083)
			−0.140	0.3	94.6%	−0.144 (−0.391, 0.100)
	T1+M3	No	−0.169	0.1	94%	−0.176 (−0.518, 0.164)
			−0.143	0.3	94.6%	−0.139 (−0.467, 0.186)

### 4.2 Approximate Balancing and Algorithm 2 Evaluation

Now we consider settings that Algorithm 2 needs to be invoked due to infeasibility of perturbed Equation (4), in particular in smaller sample size settings with noisy covariates. We set the total sample size $n=n_{s}+n_{t}=400$ with bootstrap iteration B=800, and generate covariates $X=(X_{1},\ldots ,X_{5})$ from uniform distribution U(−2,6). The rest of the settings are the same as in the previous subsection.

The performance of our methods is summarized in Table 3. We also report the percent of non‐feasible solutions over M×B iterations for exact balancing. In terms of bias evaluation, for exact balancing, even though some simulations may not admit solutions, the bias of ${\hat{\tau }}_{w}$ is small when the consistency conditions are met. When we use approximate balancing for target ATE estimation simulation cases with no exact balancing solution, the bias is larger as it trades bias for variance. In terms of the CI construction, when most of the perturbations admit an exact balancing solution, our findings about the CI coverage are consistent with what we observe in Table 1 and Table 2. Especially, when the consistency conditions are satisfied, we find that both exact and approximate balancing CI could cover $\tau^{*}$ around 95% times. However, regardless of the consistency conditions, if we could not admit enough feasible solutions during the CI construction process, we would get poor CI coverage for exact balancing. The CI constructed by the approximate balancing method is wider and thus could help in this situation with a better coverage.

**TABLE 3:** Empirical evaluation for target ATE estimation and CI coverage using RPM‐CI and RPM‐AB methods.

Settings		Consistency Condition	$\tau^{*}$	Methods	% Infeasible	Empirical coverage $\tau^{*}$	Average ${\hat{\tau }}_{w}$ (95% CI)
Linear	T1+M2	(b)	−0.652	RPM‐CI	26.0%	87.3%	−0.660 (−1.335, 0.125)
				RPM‐AB	0%	92.6%	−0.624 (−1.414, 1.174)
	T2+M1	No	0.800	RPM‐CI	26.9%	60.9%	−0.017 (−1.029, 1.075)
				RPM‐AB	0%	90.2%	0.005 (−0.958, 1.890)
Nonlinear	T1+M1	(a)	0.038	RPM‐CI	5.6%	95.2%	0.034 (−0.702, 0.767)
				RPM‐AB	0%	94.6%	0.030 (−0.806, 0.808)
	T2+M2	No	0.539	RPM‐CI	4.9%	70.4%	1.083 (0.256, 2.036)
				RPM‐AB	0%	74.2%	1.095 (0.223, 2.212)

## 5 Evaluation With a Real Data Setting

In this section, we illustrate the proposed method by evaluating and generalizing the causal effect of transthoracic echocardiography (TTE) on 28‐day survival, using data from the MIMIC‐III database [27]. The study population includes 6361 ICU patients, of whom 51.3% underwent TTE either during or within 24 h before ICU admission. Following Chen et al. [1], we restrict the dataset to the first ICU admission and exclude patients from coronary and cardiac surgical units. Our goal is to estimate the effect of TTE in surgical ICU (SICU) patients and generalize it to medical ICU (MICU) patients with sepsis incorporating uncertainty through CI construction and evaluation. In appendix, we also provide a cross‐validation based evaluation using the same data base.

TTE is a fundamental, noninvasive modality for hemodynamic assessment in critically ill patients, providing timely information to guide fluid management, vasoactive therapy, and diagnosis of cardiac dysfunction. Early use of TTE, either during or within 24 h before ICU admission, has the potential to guide timely diagnostic and therapeutic decisions, which may directly influence short‐term outcomes such as 28‐day survival [28]. However, its prognostic impact may vary by ICU setting. SICU and MICU populations are different in terms of the patients' physiological status, and clinical management. Compared with SICU patients, MICU patients are typically older, have more physiologic derangements and comorbidity burden, whereas SICU patients more often present with acute postoperative derangements [29]. These systematic differences in patient characteristics, underlying causes of hemodynamic instability, and therapeutic priorities suggest that the survival benefit of early TTE may not be uniform across ICU settings. This underscores the need to account for population heterogeneity when evaluating and generalizing treatment effects across SICU and MICU subgroups.

The dataset we used encompasses demographic details, such as age, gender, and weight, along with severity at admission measured by the Simplified Acute Physiology Score (SAPS), Sequential Organ Failure Assessment (SOFA) score, and Elixhauser comorbidity score. Additionally, it includes comorbidity indicators (denoted as ${\text{cmb}}_{i}$), including congestive heart failure (CHF), atrial fibrillation (AFib), respiratory failure (RF), and malignant tumor. Vital signs like mean arterial pressure, heart rate, and temperature, as well as laboratory results, are also part of the dataset. To address right‐skewed distributions of lab results, a log transformation is applied, and standardization is employed for continuous variables. Missing values are addressed through imputation using the missForest method, which is a flexible non‐parametric missing value imputation approach with no assumptions needed [30].

Table 4 summarizes baseline characteristics for both ICU types and compares TTE and non‐TTE groups with their Standardized Mean Difference (SMD). Of the 6361 patients, 1364 were admitted to the SICU (with 669 receiving TTE) and 4997 to the MICU (with 2593 receiving TTE). Compared to MICU patients, SICU patients were slightly younger and had higher mean arterial pressure and partial pressure of oxygen values, but lower heart rates and lower severity scores at admission (including SOFA and Elixhauser comorbidity scores). SICU patients were also less likely to have a history of CHF or malignant tumor. When comparing patients who received TTE to those who did not, TTE recipients had larger body weight and higher rates of comorbidities including CHF, RF, and AFib. They also presented with higher severity at admission including SOFA and SAPS.

**TABLE 4:** Summary statistics of baseline characteristics.

	SICU (N=1364)	MICU (N=4997)	SMD	TTE (N=3262)	non‐TTE (N=3099)	SMD
Age	64.79 (16.28)	66.59 (17.02)	−0.111^a^	65.74 (16.55)	66.69 (17.21)	−0.057
Weight	80.75 (25.18)	81.00 (25.71)	−0.010	83.28 (27.08)	78.49 (23.69)	0.177^a^
Female	674 (49%)	2459 (49%)	0.004	1558 (48%)	1575 (51%)	−0.061
Body Temperature	36.83 (1.97)	36.78 (1.40)	0.023	36.85 (1.89)	36.74 (1.06)	0.057
Mean Arterial Pressure	83.78 (19.82)	78.83 (19.42)	0.250^a^	79.97 (19.87)	79.80 (19.33)	0.009
Heart Rate	90.62 (20.26)	94.90 (20.90)	−0.212^a^	94.95 (21.73)	92.96 (19.81)	0.092
Platelet	5.23 (0.62)	5.13 (0.72)	0.160^a^	5.13 (0.71)	5.18 (0.69)	−0.068
Partial Pressure of Oxygen	4.90 (0.63)	4.60 (0.62)	0.480^a^	4.66 (0.63)	4.68 (0.64)	−0.028
Lactate	1.10 (0.43)	1.10 (0.44)	0.016	1.10 (0.43)	1.10 (0.44)	−0.009
Blood Urea Nitrogen	3.10 (0.67)	3.35 (0.71)	−0.365^a^	3.36 (0.70)	3.22 (0.71)	0.195^a^
SAPS	19.75 (5.30)	20.34 (5.72)	−0.111^a^	20.76 (5.44)	19.63 (5.79)	0.208^a^
SOFA	5.14 (3.60)	6.02 (3.76)	−0.244^a^	6.33 (3.79)	5.30 (3.62)	0.271^a^
Elixhauser Score	8.17 (7.60)	9.62 (7.57)	−0.191^a^	10.07 (7.67)	8.51 (7.45)	0.204^a^
CHF	306 (22%)	1561 (31%)	−0.211^a^	1304 (40%)	563 (18%)	0.445^a^
AFib	370 (27%)	1308 (26%)	0.021	1056 (32%)	622 (20%)	0.263^a^
RF	700 (51%)	2782 (56%)	−0.087	2127 (65%)	1355 (44%)	0.451^a^
Malignancy tumor	258 (19%)	1253 (25%)	−0.157^a^	729 (22%)	782 (25%)	−0.069

*Note:* Results are presented as Mean (SD); *n* (%).

^a^ SMD larger than 0.1.

**FIGURE 1:**
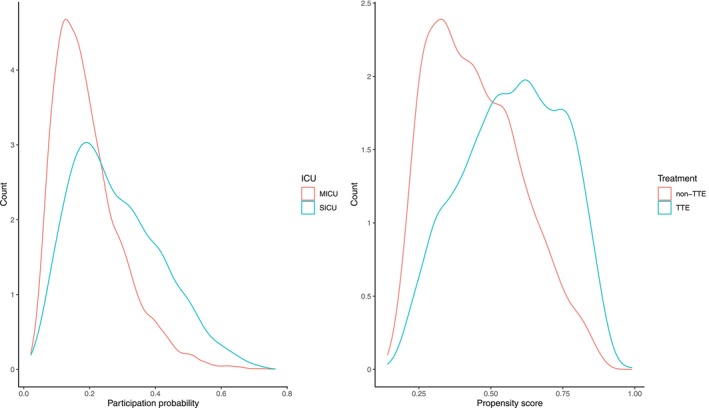
Density plots for the estimated participation probability (left panel), and the estimated propensity score (right panel).

To conservatively account for potential covariate shift between source (SICU) and target (MICU), we select all variables except weight, RF and AFib for $K_{h}$, which we assume are collected for both SICU and MICU. We select weight, RF and AFib as the additional confounder set $K_{g}$ that further correct for imbalances between TTE and non‐TTE groups within the SICU sample, as they are important confounders but not significantly different between SICU and MICU from Table 4. Since individual level data of receiving TTE and ICU type were fully observed, Figure 1 visualizes the estimated participation score $\left(\hat{\rho }(X)\right)$ and propensity scores $\left(\hat{\pi }(X)\right)$ to verify sufficient overlap between SICU and MICU and between TTE and non‐TTE samples. Both scores were estimated using logistic regression models accounting for all baseline covariates. This diagnostic assessment confirms the validity of the positivity assumptions (Assumptions 3 and 5), demonstrating adequate overlap for causal generalization.

Using entropy balancing, the estimated ATE of TTE for SICU patients is 0.029 (95% CI: [−0.014, 0.072]), while the ATE for MICU patients is 0.063 (95% CI: [0.040, 0.087]). Applying causal generalization to transport the effect from SICU to MICU, we utilize only summary level information (mean and variance) from MICU patients. The causally generalized ATE estimate is 0.053 (95% CI: [−0.007, 0.108]). Although this generalized estimate does not reach statistical significance, it shows an increased effect relative to the original SICU estimate and is directionally consistent with the MICU ATE. The slightly wider confidence interval may reflect the larger variability introduced by the relatively smaller SICU sample size or by higher‐order moment differences in covariate distributions between SICU and MICU populations. Collectively, these patterns suggest the presence of covariate shift, indicating that ICU‐specific factors may influence the magnitude of TTE's effect on 28‐day survival. These findings highlight the importance of considering population heterogeneity when generalizing treatment effects across ICU types.

## 6 Discussion and Conclusion

We have developed a resampling‐based perturbation method for CI construction to make inference about generalizing ATE estimation to a target population. It is an important step to complement the work of Chen et al. [1] to quantify the uncertainty associated with the estimated treatment effect for the target population. Although we require slightly more information from a target sample than Chen et al. [1] did, our requirement is minimum as we only need the variance of the summary statistics ${\bar{H}}_{𝒯}$. Note that for binary and discrete variables, such variance is not needed as we can directly use ${\bar{H}}_{𝒯}$ to estimate its variance. When the target sample's individual data is available but cannot be shared due to privacy reasons, then requesting this further information is relatively straightforward.

To achieve an unbiased causal generalization, exact balancing is essential, as it ensures that covariates are equally balanced between populations. For the CI construction using the resampling‐based perturbation method, exact balancing should be prioritized because it directly aligns with the goal of unbiased causal generalization by precisely matching covariate distributions. However, when a feasible solution for exact balancing is unattainable due to sample size limitations or high‐dimensional covariates, approximate balancing can be a practical alternative. Although it may introduce a small bias, approximate balancing provides a close solution that maintains the integrity of the analysis by minimizing discrepancies in covariate distributions. Therefore, we recommend approximate balancing only as a secondary option, to be used when exact balancing solutions are not feasible.

## Funding

This work was supported by the Patient‐Centered Outcomes Research Institute (Grant No. ME‐2024C1‐37433) and the National Science Foundation (Grant No. DMS‐2515263).

## Conflicts of Interest

The authors declare no conflicts of interest.

## Figures and Tables

**FIGURE B1:**
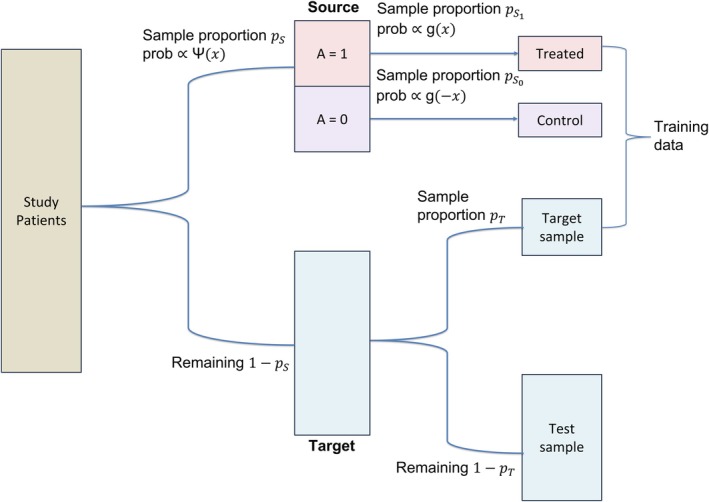
CV‐based evaluation workflow in a real setting.

## Data Availability

The data that support the findings in this paper were derived from the following resources available in the public domain: MIMIC‐III Clinical Database Version1.4 (https://physionet.org/content/mimiciii/1.4/). An R package for the method in this paper is available on GitHub: https://github.com/yc702/EBalGen, which includes the simulation code for the paper and a readme file with instructions about installing the package and using the package functions.

## Appendix A Proof of Identification

We show that $\tau^{*}=𝔼\{Y(1)-Y(0)|S=0\}$ can be identified with observed data under Assumption 1‐5 by showing the following with ρ=𝔼{ρ(X)}=ℙ(S=1):

$$\begin{array}{ll} & 𝔼\{Y(1)-Y(0)|S=0\}\\ & \;=𝔼\left[\frac{1}{1-\rho }\frac{1-\rho (X)}{\rho (X)}S\left\{\frac{AY}{\pi (X)}+\frac{(1-A)Y}{1-\pi (X)}\right\}\right] \end{array}$$

This equation follows from

(A1)
$$\begin{array}{ll} & 𝔼[\frac{1}{1-\rho }\frac{1-\rho (X)}{\rho (X)}S\left\{\frac{AY}{\pi (X)}-\frac{(1-A)Y}{1-\pi (X)}\right\}]\\ & \;=\frac{1}{1-\rho }𝔼\{\frac{1-\rho (X)}{\rho (X)}𝔼[S\{\frac{AY}{\pi (X)}-\frac{(1-A)Y}{1-\pi (X)}\}|X,S]\}\\ & \;=\frac{1}{1-\rho }𝔼(\{1-\rho (X)\}𝔼[S\{\frac{AY}{\pi (X)}+\frac{(1-A)Y}{1-\pi (X)}\}|X,S=1]) \end{array}$$

(A2)
$$\begin{array}{ll} & \;=\frac{1}{1-\rho }𝔼[\{1-\rho (X)\}𝔼\left\{Y(1)-Y(0)|X,S=1\right\}] \end{array}$$

(A3)
$$\begin{array}{ll} & \;=\frac{1}{1-\rho }𝔼[\{1-\rho (X)\}𝔼\left\{Y(1)-Y(0)|X,S=0\right\}] \end{array}$$

(A4)
$$\begin{array}{ll} & \;=\frac{1}{1-\rho }𝔼[𝔼(1-S|X)𝔼\left\{Y(1)-Y(0)|X,S=0\right\}] \end{array}$$

$$\begin{array}{ll} & \;=\frac{1}{1-\rho }𝔼(𝔼[(1-S)𝔼\{Y(1)-Y(0)|X,S=0\}|X])\\ & \;=\frac{1}{\mathbb{P}(S=0)}𝔼[(1-S)𝔼\{Y(1)-Y(0)|X,S=0\}]\\ & \;=𝔼[𝔼\{Y(1)-Y(0)|X,S=0\}|S=0]\\ & \;=𝔼\{Y(1)-Y(0)|S=0\} \end{array}$$

with our weights $\left\{w_{i}:i\in 𝒮\right\}$ takes form of $w_{i}=\left\{\frac{A_{i}}{\pi (X_{i})}+\frac{1-A_{i}}{1-\pi (X_{i})}\right\}\frac{E(S_{i})(1-\rho (X_{i}))}{\left(1-E(S_{i}))\rho (X_{i}\right)}$.

(A1) and (A4) follow from the definition of ρ(X). (A2) follows from Assumption 2 the definition of π(X). (A3) follows Assumption 4.

## Appendix B Cross‐Validation Based Evaluation With a Real Data Setting

Here, we use the same real data setting as above derived from the MIMIC‐III database [27], but we would like to evaluate the validity of the constructed CI. Unlike simulation settings where the true target average treatment effect (ATE) is known, the true causal effect in this observational context is unknown. Therefore, we additionally use this application to assess the empirical validity of the proposed CI construction method when applied to real‐world data. To address the unknown true effect, we adopt a cross‐validation (CV) based strategy described below, which allows us to evaluate the stability and performance of the interval estimates across multiple data splits. In particular, we partition the data set into subsets, train the model on some of these subsets, and then evaluate its performance on the remaining subset. This not only illustrates the practical utility of the method in a clinically relevant setting but also provides evidence on how well the proposed inference procedure performs when the ground truth is not observable, as is typical in medical observational studies.

Figure B1 is a diagram showing the whole workflow of our CV‐based evaluation. For each round of CV, we first partition $p_{S}$ proportion of the total study population into the source population, with the remainder as the target population. The partition or sampling probability is in proportion to a function Ψ, which includes some important effect modifiers. Then, within the source population, we generate source samples using a function g, which includes some key confounding factors. In particular, $p_{S_{1}}$ proportion of the treated and $p_{S_{0}}$ of the control populations become treated and control samples. Next, we randomly split the target population with $p_{T}$ proportion into target and the rest into test samples. Suppose in the target sample, we only know the summary level information while in the test sample we know all the individual level information. The above steps will be repeated many times and the source treated, control, and target samples will be used as the training data to estimate target population treatment effect and CI. To obtain an oracle estimate of the target population ATE, we repeat the splitting procedures and use the test sample to construct a CV‐based treatment effect $\tau_{cv}^{*}$ as our oracle estimator. We will use this $\tau_{cv}^{*}$ as the benchmark to evaluate the bias and CI of the target population treatment effect. The rest of the section illustrates the details of this CV‐based procedure used in our real‐setting.

To comprehensively assess across diverse scenarios, we manipulate various degrees of confounding and covariate shift while keeping the covariate‐outcome relationship unchanged in the actual data. Initially, we select $p_{S}=40\%$ of the entire dataset with probability proportional to Ψ(x) to form the source population. The remaining data is then randomly divided into a target sample, with a $p_{T}=1/3$ probability, and a test sample. The probability of being selected as a source sample is proportional to

$$\begin{array}{ll} & \Psi \left\{\kappa_{𝒮}(-0.3\times \text{age}+0.3\times {\text{cmb}}_{1}+0.4\times {\text{cmb}}_{2}+0.3\times {\text{cmb}}_{3}\right.\\ & \;+\left.0.4\times {\text{cmb}}_{4}-0.5)\right\}. \end{array}$$

where Ψ(x)=0.8Φ(x)+0.1 with Φ(x) being the standard normal CDF. Here, $\kappa_{𝒮}$ is a parameter to reflect different levels of covariate shift, where $\kappa_{𝒮}$ equals 1 for small covariate shift and 5 for large covariate shift. Under this sampling design, the source population is younger and more likely to have comorbidities than the target population. Among the source samples, we randomly select $p_{S_{1}}=1/2$ of the TTE patients and $p_{S_{0}}=1/2$ of the non‐TTE patients to form the source sample. The TTE patients are selected with probability proportional to g(x) while the non‐TTE patients are selected with probability proportional to g(−x) with

$$\begin{array}{l} g(x)=\Psi \{\kappa_{𝒜}(0.3\times \text{SAPS}+0.4\times \text{SOFA}-0.5\times \text{Elixhauser})\}\;. \end{array}$$

We consider two choices of $\kappa_{𝒜}$ in g: (a) $\kappa_{𝒜}=0$, so all the patients in this step are sampled with equal probability; (b) $\kappa_{𝒜}=1$, which induces additional confounding determined by a linear combination of the severity scores.

In total, we have four settings. Under each one, we run M=500 times replications of the above CV procedures and B=1000 perturbations using the exact balancing method to construct the estimator ${\hat{\tau }}_{cv}$ and its confidence intervals. We assume that the target sample only includes information about the average and variance of the demographic covariates and comorbidity indicators. The oracle estimate of the target population ATE $\tau_{cv}^{*}$ is estimated using the entropy balancing weighting method incorporating full information in the test data. To be more specific, we repeat the above CV procedures 8000 times and get the estimated target ATE. The average of it would be our oracle estimator of the target population ATE $\tau_{cv}^{*}$. The CV splitting set‐up here makes a good overlap between source and target samples. Thus, we did not encounter the infeasibility issue for exact balancing.

For evaluation, we examine the bias and percentage of times the constructed CI covers the oracle estimator $\tau_{cv}^{*}$. Table B1 summarizes $\tau_{cv}^{*}$ empirical coverage percentage and the estimator ${\hat{\tau }}_{cv}$ under each scenario. As we can see, the proposed method could cover the oracle estimator around 95% of the time. In evaluating bias, we find that the bias of empirical ${\hat{\tau }}_{cv}$ is ignorable under different settings.

**TABLE B1:** Target ATE Estimation and CI Coverage in a Real Setting.

Setting				
Confounding	Covariate shift	$\tau_{cv}^{*}$	Empirical coverage of $\tau_{cv}^{*}$	Average ${\hat{\tau }}_{cv}$ (95% CI)
Extra $\left(\kappa_{A}=1\right)$	Small $\left(\kappa_{s}=1\right)$	0.051	96.4%	0.048 (−0.006, 0.102)
No $\left(\kappa_{A}=0\right)$	Small $\left(\kappa_{s}=1\right)$	0.050	96%	0.052 (0.001, 0.104)
Extra $\left(\kappa_{A}=1\right)$	Big $\left(\kappa_{s}=5\right)$	0.049	95.6%	0.052 (−0.005, 0.107)
No $\left(\kappa_{A}=0\right)$	Big $\left(\kappa_{s}=5\right)$	0.049	95.8%	0.054 (0.001, 0.107)
